# Mathematics emotion profiles: stability and change during Grades 7 and 8

**DOI:** 10.1007/s10212-025-00972-4

**Published:** 2025-06-11

**Authors:** Tanja Held, Tina Hascher

**Affiliations:** 1 School of Education, Liverpool John Moores University, Liverpool, Great Britain UK; 2 Department of Research in School and Learning, Institute of Educational Science, University of Bern, Bern, Switzerland

**Keywords:** Emotion profiles, Intervention, Mathematics, Patterns of change

## Abstract

Academic emotions are not mutually exclusive, and students can experience various emotions simultaneously. We aimed at identifying distinct emotion profiles in mathematics in the lowest-ability tier in lower secondary school. Also, we investigated the patterns of change to students’ mathematics emotion profiles in Grades 7 and 8, and whether an intervention setting impacted these patterns of change compared to the control setting. Latent profile analysis (LPA) and random intercept latent transition analysis (RI-LTA) were applied to assess the mathematics emotion profiles and the probability of transitioning between the profiles of 348 students. Results revealed three emotion profiles: a *mixed emotion profile*, a *rather positive emotion profile*, and a *predominantly positive emotion profile* with a high level of stability across Grades 7 and 8. The three mathematics emotion profiles can be predicted by gender and mathematics achievement. Finally, RI-LTA revealed that the intervention had different effects on the mathematics emotion profiles.

**Supplementary Information:**

The online version contains supplementary material available at 10.1007/s10212-025-00972-4.

Students experience a wide range of different emotions in their everyday school lives. Existing research has specifically emphasized the importance of achievement emotions for learning, academic achievement, and well-being (Pekrun & Linnenbrink-Garcia, 2014). Positive emotions such as enjoyment are positively related to higher academic achievement, motivation, and well-being, while negative emotions such as anxiety tend to be negatively related (Camacho-Morles et al., 2021; Pekrun et al., 2017). However, emotional experiences are not mutually exclusive, and students may experience multiple academic emotions at the same time, which can be explored using a person-centered approach (Fernando et al., 2014; Larsen et al., 2007). Investigating co-occurring emotions through a person-centered research approach may provide new perspectives that help to understand the complexity of students’ emotional experiences at school. Experiencing positive and negative emotions simultaneously may reveal different effects than experiencing exclusively positive or negative emotions, and the relationships between those differences in emotional experiences and outcomes may be strengthened, weakened, or qualitatively altered (Barker et al., 2016; Robinson et al., 2017). However, prior research focused predominantly on the development of single emotions, while far less is known about the stability and change of emotion combinations as expressed in emotion profiles. Based on the assumed interaction between different emotions, the development of emotion profiles should also be investigated. Finally, applied to intervention research, a person-centered approach can help researchers discover the effects of an intervention on different subgroups by identifying individuals with common (emotional) characteristics and assuming heterogeneous effects on intervention outcomes (Laursen & Hoff, 2006).

Prior research has shown that academic emotions are domain-specific and thus differ across domains (e.g., Goetz et al., 2007). The present study focuses on emotions specific to the domain of mathematics among students in the lowest-ability tier (at-risk students) in lower secondary education. We examine student emotions in Grades 7 and 8 because existing research indicates that this period is characterized by a decline in positive emotions and an increase in negative emotions, particularly in mathematics learning (e.g., Meyer & Schlesier, 2021; Vierhaus et al., 2016), making this stage a particularly relevant period for investigating emotional trajectories and intervention effects. Specifically, the study aims to (1) identify subpopulations of students with distinct mathematics-related emotion profiles, (2) examine gender and mathematics test scores as predictors of these profiles, (3) explore the stability and change of students’ mathematics emotion profiles from the beginning of Grade 7 to the end of Grade 8, and (4) analyze whether an intervention program (workshops with the aim of promoting positive mathematics emotions and learning motivation) influences the patterns of change in emotion profiles compared to the control setting.

## Students’ emotions in education

Learning is not only a cognitive but also an emotional endeavor. In the present study, we adopt the control-value theory (Pekrun, 2006) as the conceptual framework for analyzing achievement emotions in mathematics learning. Achievement emotions are defined as emotions that arise in learning situations or in response to academic success or failure (Pekrun, 2006). This theory provides a structured approach to understanding how achievement emotions are linked to students’ perceptions of control over their learning and the value they attribute to academic tasks. While also broader constructs such as affect (Hannula, 2012) or attitudes (Di Martino & Zan, 2015) have been used to examine students’ emotional relationships with mathematics learning, achievement emotions provide a more precise lens for investigating students’ general responses over time that directly impact their learning outcomes.

Achievement emotions are linked with important predictors of academic success, such as motivation and learning behavior (Pekrun, 2006, 2014). Positive achievement emotions, such as enjoyment, which is a positive, activating, activity-related emotion, and pride as an outcome-related emotion, enhance learning and achievement. In contrast, negative achievement emotions like anger, anxiety, and boredom generally have unfavorable effects on learning and achievement (Camacho-Morles et al., 2021; Ranellucci et al., 2015). Anger is an activating, activity-related emotion, while anxiety is an activating, outcome-related emotion. Boredom is a deactivating, activity-related emotion commonly encountered in everyday school settings (Pekrun, 2006). In mathematics education, emotions play a crucial role in shaping students’ engagement and problem-solving strategies (Gómez-Chacón, 2015; McLeod, 1989). The emotional trajectories are thereby shaped by past experiences (Hannula, 2012) and embedded in the instructional context, including mathematical task design and the role of teachers’ affective support (Chen & Leung, 2015).

Several longitudinal studies have examined the development of students’ emotions (e.g., Meyer & Schlesier, 2021). Existing research across different subjects indicates a decline in positive emotions specifically during secondary education (Bieg et al., 2019; Meyer & Schlesier, 2021). In contrast, negative emotions (e.g., anger, anxiety, boredom) tend to increase during this period (Raccanello et al., 2013; Vierhaus et al., 2016). These emotional shifts are particularly pronounced in mathematics learning (Raccanello et al., 2013), where affective experiences are strongly tied to students’ self-efficacy and their perceptions of mathematical competence (Van der Beek et al., 2017).

In academic settings, students may experience multiple achievement emotions with varying levels of intensity at the same time. Accordingly, several studies have reported the co-occurrence of achievement emotions (e.g., Jarrell et al., 2016; Tze et al., 2022), and the relationships between achievement emotions and outcomes may be amplified, attenuated, or qualitatively altered when different emotions co-occur (Barker et al., 2016; Robinson et al., 2017). Thus, investigating multiple emotional experiences can contribute to the understanding of how emotions impact academic outcomes. Identifying the co-occurrence of achievement emotions may also be of particular importance for understanding the specific needs of the individual student (Robinson et al., 2020). In the mathematics classroom, students’ achievement emotions are not isolated phenomena, but are embedded in a broader socio-cultural and instructional context that shapes their expression (Radford, 2015). However, research on the co-occurrence of achievement emotions in education and, more concretely, the mathematics classroom, is still scarce. Moreover, little is known whether gender and achievement may predict this co-occurrence during adolescence.

### Emotion profiles in mathematics

Studies of students’ achievement emotions traditionally follow a variable-centered approach by analyzing single discrete emotions such as enjoyment, anger, or boredom (e.g., Putwain et al., 2018). However, some recent person-centered studies in education have shed light on the existence of different emotion profiles in science and mathematics in different student groups by analyzing several discrete emotional states or traits simultaneously (e.g., Ganotice et al., 2016; Jarrell et al., 2017). Although the results are not directly comparable due to differences in age groups or measures, they can provide important information regarding which emotion profiles to expect in secondary education. Existing research found two to four different profiles, which differ in their composition of different emotions (for an overview of studies using person-centered approaches to examine academic emotions, see Karamarkovich & Rutherford, 2021). Most studies in education across different age groups have found a positive emotion profile, characterized by higher levels of various positive emotions than negative emotions, and a negative emotion profile showing higher levels of negative emotions than positive emotions (Robinson et al., 2020). However, some studies have found a mixed emotion profile with similar levels of co-occurring positive and negative emotions (e.g., Abd-El-Fattah, 2018; Karamarkovich & Rutherford, 2021; Tze et al., 2022).

Existing research on the links between academic achievement and achievement emotion profiles revealed inconsistent results: For example, Jarrell et al. (2016) found no significant relationship between prior achievement and emotion profiles among university students in science, while Karamarkovich and Rutherford (2021) reported significant differences among primary school students in mathematics. Students with a negative emotion profile had significantly lower prior mathematics achievement scores than students with a positive emotion profile. Thus, further research is needed to gain insights into how achievement is related to emotion profiles in secondary education. In addition, while prior research has examined gender differences in mathematics-related emotions (e.g., Frenzel et al., 2007a; OECD, 2014), findings have been inconsistent, particularly in studies using person-centered approaches. Some studies reported no significant differences (Robinson et al., 2017), whereas others suggested gender-specific differences in emotion profiles (Karamarkovich & Rutherford, 2021). Given that our study focuses specifically on students in the lowest-ability tier, further research is needed to understand how gender and achievement emotions interact within this student group.

Overall, research on emotion profiles in education is scarce (Karamarkovich & Rutherford, 2021), and several questions are still unanswered, such as the association of emotion profiles with prior achievement and gender in secondary education, the prevalence of the profiles among students in the lowest-ability tier, or the responsiveness of students in different emotion profiles to emotion interventions. In addition, most prior studies have examined emotion profiles using a cross-sectional approach, with the exception of Tze et al. (2022), who investigated the stability of emotion profiles in an Introductory Psychology course over 6 months. However, less is known about emotion profile stability and changes in other age groups or longer time spans.

### Emotion interventions in mathematics

The unfavorable decrease of positive emotions in mathematics learning in adolescence (e.g., Meyer & Schlesier, 2021) in combination with their high relevance for learning success suggests implementing measures to help counteract this development and test their efficacy. The results of the few existing emotion intervention studies demonstrate that students’ mathematics emotions can be influenced (e.g., Gläser-Zikuda et al., 2005). Most of these interventions targeting emotions belong to the social-psychological interventions. These interventions aim to ameliorate students’ feelings, thoughts, and beliefs in and about learning (Walton & Wilson, 2018). These interventions are therefore not about teaching content or skills, but rather about triggering psychological processes that can change perceptions and attitudes in mathematics learning (Yeager & Walton, 2011). Existing research also indicates that mathematical task design and teacher support can influence students’ emotional engagement (Chen & Leung, 2015; Lei et al., 2018). Thus, an intervention that integrates meaningful activities related to mathematics, promotes autonomy, and fosters a positive learning climate may have stronger and more lasting effects on students’ emotions than interventions that aim at enhancing specific mathematical skills.

However, most interventions seem to have weak effects, which is often explained by the heterogeneity of students’ emotional development (e.g., Hamm et al., 2014). For example, students with low levels of positive emotions in mathematics might benefit from an intervention, while students who already have high levels of positive emotions might benefit less or even face negative side effects (Walton & Wilson, 2018). Based on these differential effects, we suggest considering that students’ emotional characteristics in mathematics, as represented by emotion profiles, may influence the effectiveness of an intervention. Thus, a person-centered perspective might help to improve our understanding of student emotions and provide insights into the impact of interventions.

## The present study

Prior research on student emotions following a person-centered approach has shown promising results, as it has suggested the co-existence of various achievement emotions and identified distinct emotion profiles among students. However, little is known about these emotional profiles in students in the lowest-ability tier in lower secondary education (Grades 7–9), a group that has experienced negative academic selection based on their prior achievement. Furthermore, research has yet to examine how these profiles evolve over time and whether interventions can influence their trajectories. The present study aims to address these gaps by examining the emotional experiences in this student group and assessing the impact of a social-psychological intervention designed to foster positive emotions and motivation in mathematics learning.

First, we investigate the mathematics emotion profiles of students in the lowest-ability tier in lower secondary education based on the emotions of *enjoyment*, *pride*, *anger*, *anxiety*, and *boredom*. These emotions occur frequently in mathematics instruction (e.g., Frenzel et al., 2007b), and together, they cover a range of different emotions in everyday school life. Using latent profile analysis (LPA), we investigate whether distinct mathematics emotion profiles emerge in this student group, as observed in previous research for other groups (e.g., Karamarkovich & Rutherford, 2021). Additionally, we explore the role of gender and prior mathematics achievement in predicting students’ emotion profiles, as previous findings in person-centered studies have been inconsistent (e.g., Hanin & Van Nieuwenhoven, 2019; Robinson et al., 2017). Since students in the lowest-ability tier often experience repeated academic struggles from primary school onward, it is plausible that lower achievement may be associated with more negative emotion profiles.

Second, we investigate the development of the emotion profiles over time by applying random intercept latent transition analysis (RI-LTA). Given that the transition to secondary education initiates a new academic phase for the students (Held & Hascher, 2023a), this allows us to analyze whether their mathematics emotion profiles remain stable or change over their first two years in lower secondary education. While variable-centered research suggests a general decline in positive emotions over time (e.g., Hagenauer & Hascher, 2010; Meyer & Schlesier, 2021; Vierhaus et al., 2016), person-centered studies on profile transitions remain scarce. Based on prior findings with university students (Tze et al., 2022), we expect that most emotion profiles will remain stable, but when changes occur, they will likely involve a shift from profiles with higher positive emotions to those with lower positive emotions.

Third, we want to investigate whether a potentially negative change of students’ emotional profiles in lower secondary education can be tempered, and analyze the effects of a social-psychological intervention designed to promote positive emotions and motivation in mathematic learning. We apply RI-LTA to investigate whether the intervention influenced students’ emotion profile trajectories over time and whether its effects differed across emotion profiles. While a previous variable-centered analysis of our data found no significant average intervention effect (Held & Hascher, 2022), it remains unclear whether specific subgroups benefited from the intervention, while others did not, blurring the overall effects. For example, reflecting on the importance of mathematics may have a positive effect on students with a negative emotion profile and promote positive emotions because the value of learning mathematics increases, while students with a positive emotion profile may feel additional pressure and experience negative emotions (e.g., anxiety) due to the additional focus that is given to the importance of mathematics. Therefore, we aim to investigate whether there are differential effects in terms of emotion profiles that were not detected in the variable-centered analysis.

Overall, this study provides a comprehensive perspective on mathematics emotion profiles by examining their formation, development over time, and responsiveness to an intervention. By considering the trajectories of emotion profiles and the potential for interventions to influence these trajectories, we offer a nuanced understanding of how students in the lowest-ability tier experience emotions in mathematics.

## Method

### Participants

The data used in this study stemmed from the “Maintaining and Fostering Students’ Positive Learning Emotions and Learning Motivation in Maths Instruction During Adolescence” intervention project in lower secondary education, which was funded by the Swiss National Science Foundation (Grant Number 156710). By this funding, the study received full ethical consent. The ethical principles of the APA were followed. Participation was voluntary, and participating students were required to have parental or guardian consent for participation. The study took place in the German-speaking part of the canton of Bern in Switzerland and employed a quasi-experimental design with two intervention groups (student–teacher and student–only) and one control group. To facilitate participation and to minimize potential attrition, teachers were given the option to select in which setting they preferred to participate with their mathematics classes (*n*_student-teacher intervention_ = 8; *n*_student intervention_ = 8). Since all teachers volunteered to be part of the intervention groups, additional mathematics teachers teaching the same ability tier and grade level in either the same school or a school within the same district were recruited separately as a control group (*n* = 6). To ensure comparability, we examined the school social index, which is based on the proportion of non-Swiss students, unemployment rates, the prevalence of buildings with low residential use, and levels of sedentariness. The comparison revealed no significant differences between the groups (*M*_student-teacher_ = 1.24, range = 1.08–1.48; *M*_student intervention_ = 1.41, range = 1.12–1.66; *M*_control group_ = 1.31, range = 1.03–1.55; *F*(2,19) = 2.36, *p* = 0.121, *η*^2^ = 0.01). Additionally, teachers across all three groups (two intervention groups and the control group) did not differ regarding their self-reported enjoyment of teaching mathematics (see Brandenberger et al., 2018). The study had three measurement points throughout Grades 7 and 8. The primary objectives of the intervention were to enhance students’ motivation and positive emotions towards mathematics.

The study sample included 348 students in 22 classes in the lowest-ability tier in Switzerland,^1^ whose average age at the first measurement point was 12.75 (*SD* = 0.64). The sample was composed of 179 female students (51.4%) and 169 male students (48.6%). Of the entire sample, 134 participated in a joint student–teacher intervention, 122 participated in a student–only intervention, and 92 were in the control group. A change of mathematics teacher between Grade 7 and Grade 8 took place in four classes out of 22 classes and affected 59 students.

### Intervention

The students in the two intervention groups participated in eight identical workshops during regular mathematics classes during Grades 7 and 8. A detailed schedule with specific activity durations was established for each workshop, and all instructions were predetermined. The workshops were conducted by three trained project members and had been pre-tested.

**Fig. 1:**
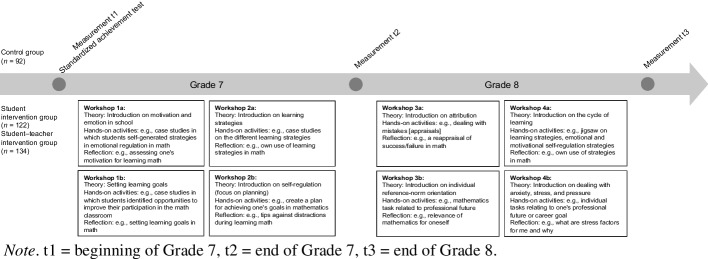
Student intervention timeline and aims. Note. t1 = beginning of Grade 7, t2 = end of Grade 7, t3 = end of Grade 8

The intervention was multi-faceted and incorporated various theoretical components of self-determination theory (Deci & Ryan, 2002), control-value theory (Pekrun, 2006), attribution theory (Weiner, 1985), and expectancy-value theory (Eccles et al., 1983). Each workshop aimed to achieve different objectives with the overarching goals of promoting learning motivation and positive mathematics emotions. The workshops were a blend of theory, hands-on activities, group collaboration, and reflection on learning and the significance of mathematics in academic learning, daily life, and students’ future careers (see Fig. 1; Brandenberger et al.,^2018^; Held & Hascher, 2023b; Sutter-Brandenberger et al., 2019). Following social-psychological interventions (see Walton & Wilson, 2018), the intervention was designed to alter students’ beliefs about their abilities, goals, and values in mathematics, thus triggering psychological processes that were expected to influence the trajectories of students’ experiences and motivational and emotional outcomes.

In the student–teacher intervention, the mathematics teachers participated in four workshops while teaching the students in Grade 7 and one year later in Grade 8. These workshops provided a theoretical background on the student workshops and aimed to expand teachers’ understanding of student emotions and motivation. Additionally, teachers collaboratively developed and refined strategies to support students’ psychological needs (need for autonomy, competence, and relatedness) and control-value cognitions in their mathematics lessons. These strategies included emphasizing the relevance of mathematics to students’ everyday lives and future careers, providing individualized feedback on progress, and integrating collaborative tasks to foster peer interactions (see also Brandenberger et al., 2018; Sutter-Brandenberger et al., 2019).

### Measures

Students’ achievement emotions (see Table 1) was assessed using a shortened version of the Achievement Emotions Questionnaire–Mathematics (AEQ–M; Pekrun et al., 2005), a validated instrument aligned with the control-value theory (Bieleke et al., 2023). All items were rated on a five-point Likert scale ranging from 1 (strongly disagree) to 5 (strongly agree). Additionally, the number of patterns was determined

**Table 1:** Scales and items

Scale and example item	Number of items	*α*_t1/t2/t3_
Enjoyment “I enjoy mathematics classes”	4	0.92/0.90/0.87
Pride “I am proud of my contributions in mathematics class”	2	0.88/0.86/0.82
Anger “Because I’m angry, I get restless in mathematics class”	4	0.82/0.83/0.82
Anxiety “I feel nervous in mathematics class”	5	0.85/0.81/0.82
Boredom “Mathematics class bores me”	3	0.76/0.83/0.82

*Note*:* t1* beginning of Grade 7, *t2* end of Grade 7, *t3* end of Grade 8

Gender (female = 0; male = 1) was included, and students’ mathematics achievement was tested during two regular consecutive mathematics lessons (90 min) at the beginning of Grade 7 using a standardized mathematics test from a national large-scale assessment. This mathematics test is aligned with the Swiss curriculum (for more information on the test, see Konsortium Mathematik, 2009).

### Statistical analyses

#### Missing data

Missing data is a prevalent issue in longitudinal studies. In the present study, missing data occurred due to the permeability between the tiers of the Swiss school system. As the study only pertains to students in the lowest-ability tier, only students who remained in such classes throughout Grade 7 were included in the analyses. At the end of Grade 8, 23% of the dependent variables were missing due to school changes, illness, out-of-school practicums, and trial apprenticeships during in-class data collection. Missing data was handled using FIML estimation in Mplus (Muthén & Muthén, 1998–2018).

#### Preliminary measurement analyses

Confirmatory factor analyses were conducted at all three measurement points to test the assumed factor structure. Model fit was adequate to good for all latent constructs. Measurement invariance was examined for all latent constructs to determine whether they were stable over time, applying a sequential testing procedure (Little, 2013). To determine the degree of measurement invariance, changes in fit indices, the comparative fit index (CFI), and the root-mean-square error of approximation (RMSEA) of the nested models were evaluated. The cut-off value for changes in CFI and RMSEA was set at less than 0.01 and 0.01–0.015 respectively (Chen, 2007).

Given the complexity of LPA and RI-LTA, factor scores were estimated using the effects coding method employed by Little et al. (2006), which reflects the observed metric of the indicators, optimally weighted by the degree to which each represents the underlying latent construct. The use of factor scores is an alternative approach that is more frequently applied in recent applications of LPA and RI-LTA (e.g., Kam et al., 2016; Morin et al., 2016).

#### Latent profile and random intercept latent transition analysis

First, time-specific LPA were estimated based on the five emotions. Model selection was based on a combination of the Bayesian information criterion (BIC), the sample size-adjusted Bayesian information criterion (aBIC), the consistent Akaike information criterion (CAIC), and the entropy value. Smaller values of BIC, aBIC, and CAIC indicate a better fit (Nylund et al., 2007), while the entropy value summarizes the quality of the classification, with a measure close to 1 indicating a good fit (Muthén, 2000). To supplement the information criteria, graphical illustrations were employed to demonstrate the benefit of additional profiles (Morin et al., 2016). Additionally, the number of patterns was determined based on the content level, taking into account the interpretability of each pattern and the number of individuals per pattern as criteria (Boscardin et al., 2008).

Second, after determining the number of profiles, the LPA solutions were integrated into a longitudinal LPA to investigate the similarity of latent profiles over time (Morin & Litalien, 2017). This step-by-step process began with verifying the number of profiles across measurement points (i.e., configural similarity). Constraints were then applied to within-profile means (i.e., structural similarity), variances (i.e., dispersion similarity), and relative size (i.e., distributional similarity; Morin & Litalien, 2017). The fit of these models was then compared using the information criteria. As suggested by Morin et al. (2016), at least two of the measures should be lower than the previous model to support the hypothesis of profile similarity. Finally, the most similar model of longitudinal LPA was converted to a RI-LTA model, which estimates the probability of transitioning between profiles at adjacent time points (Muthén & Asparouhov, 2022, p. 1).

To examine cross-sectional relations between gender and mathematics test scores, and mathematics emotion profiles, we used the three-step approach (R3STEP) to avoid changes in latent classes and consider the classification uncertainty rate for each participant (Asparouhov & Muthén, 2014). To investigate how group membership (i.e., intervention group) was related to changes in students’ mathematics emotional patterns, we included it in the RI-LTA. This allowed us to investigate its effect on the probability of transitioning between profiles over time (Asparouhov & Muthén, 2014).

## Results

Across all measurement points, the emotions showed clear correlations in the expected direction (see Supplementary Information A). Measurement invariance across time was tested for all latent constructs to control whether they were stable over time and could be compared. At least, scalar invariance was supported for all measures (see Supplementary Information B).

### Emotion profiles

Latent profile models with two to six latent classes were estimated at each time point (see Supplementary Information C). The results reveal that the BIC, aBIC, and CAIC continued to decrease with the addition of latent profiles, while the plotted results showed a plateau at three to four profiles, suggesting a three- or four-profile solution (see Supplementary Information D). Along with statistical indices, it is important to consider profile interpretability. Profiles with fewer than 25 cases should be rejected because they may have lower power, accuracy, and parsimony than larger profiles (Lubke & Neale, 2006; Spurk et al., 2020). This supports the three-profile solution, as the four-profile solution involves a very small class size (K2 = 15 students). The examination of the three-to-five profile solutions also reveals that the three-profile solution resulted in well-defined and meaningful profiles, while the addition of a fourth or fifth profile resulted in the arbitrary division of existing profiles into smaller ones. Based on the plot, the fit indices, and the interpretability of classes, the three-profile solution was selected.

Next, we explored possible changes to profile structure over time. We estimated a three-wave LPA of configural, structural, dispersion, and distributional similarity. Comparing the models revealed decreasing BIC and CAIC values, supporting the similarity of the three-profile solution across time points (see Supplementary Information C). The model of distributional similarity resulted in an increase in the value of all information criteria and was thus not supported.

**Fig. 2:**
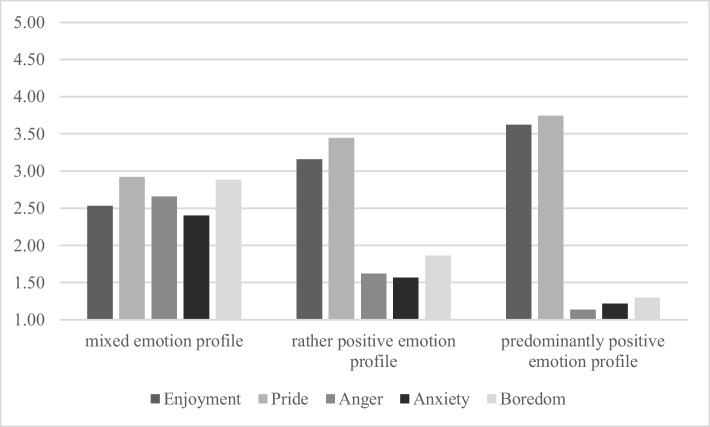
Emotion profiles

The final profile solution is depicted in Fig. 2 (mean values of profile indicator variables are reported in Supplementary Information E). The *mixed emotion profile* (*n*_t1_ = 129; *n*_t2_ = 115; *n*_t3_ = 125) was characterized by moderate scores for all five emotions. The *rather positive emotion profile* (*n*_t1_ = 152; *n*_t2_ = 118; *n*_t3_ = 148) showed moderate scores for the positive emotions and rather low scores for the negative emotions. The *predominantly positive emotion profile* (*n*_t1_ = 67; *n*_t2_ = 115; *n*_t3_ = 75) was characterized by high scores for positive emotions and very low scores for negative emotions.

### The impact of gender and mathematics achievement on emotion profiles

As indicated by the odds ratio (OR) at the beginning of Grade 7, boys—compared to girls—were significantly less likely to have the predominantly positive emotion profile than the rather positive emotion profile (*OR* = 0.34, 95% CI [0.16, 0.74]) or the mixed emotion profile (*OR* = 0.21, 95% CI [0.09, 0.48]). At the end of Grade 7, boys were significantly less likely to have the predominantly positive emotion profile than the mixed emotion profile (*OR* = 0.41, 95% CI [0.21, 0.80]). At the end of Grade 8, boys were significantly less likely to have the predominantly positive (*OR* = 0.29, 95% CI [0.13, 0.64]) or rather positive emotion profile (*OR* = 0.41, 95% CI [0.21, 0.80]) than the mixed emotion profile.

At the beginning of Grade 7, students with the rather positive (*OR* = 1.01, 95% CI [1.01, 1.02]) and predominantly positive emotion profiles (*OR* = 1.01, 95% CI [1.01, 1.01]) were significantly more likely to display high mathematics test scores than students with the mixed emotion profile.

### Stability and change of emotion profiles

The estimated transition probabilities of the final model for the three measurement points are provided in Table 2. The diagonal values in each matrix show the probability of a student retaining an emotion profile, whereas values off the diagonal represent the likelihood of changing from one profile to another. Overall, we found that emotion profile membership in adolescence is fairly stable across time, with the probabilities of retaining a profile varying between 48.4% and 77.0%. Thus, the majority of the students continued to have a specific profile over time, and having the same profile was more likely than transitioning to another profile. However, other trends can be noted: For students with the mixed emotion profile, there was a higher probability of transitioning to the rather positive (22.0% and 19.9%) than the predominantly positive emotion profile (13.6% and 3.1%). Conversely, there was a lower probability of transitioning from the predominantly positive emotion profile to the mixed emotion profile (16.1% and 7.4%) than to the rather positive emotion profile (20.7% and 44.6%).

**Table 2:** Transition probabilities for the final latent transition analysis between profiles: (a) t1 to t2 and (b) t2 to t3

a)			t2		
		Emotion profile	Mixed	Rather positive	Predominantly positive
	t1	Mixed	0.644	0.220	0.136
		Rather positive	0.145	0.484	0.371
		Predominantly positive	0.161	0.207	0.631
b)			t3		
		Emotion profile	Mixed	Rather positive	Predominantly positive
	t2	Mixed	0.770	0.199	0.031
		Rather positive	0.215	0.642	0.143
		Predominantly positive	0.074	0.446	0.479

*Note*:* t1* beginning of Grade 7, *t2* end of Grade 7, *t3* end of Grade 8

Generally, the probability of transitioning between the first and second measurement points and the second and third points was similar. However, between the first and second measurement points, the probability of transitioning from the rather positive to the predominantly positive emotion profile (37.1%) was higher than it was between the second and third measurement points (14.3%). This indicates a positive trend during Grade 7, with an increase in positive emotions and a decrease in negative emotions. This is also reflected in the increased number of students who had the predominantly positive emotion profile at the second measurement point (*n*_t2_ = 115) compared to the first measurement point (*n*_t1_ = 67). Transitioning from the rather positive to the predominantly positive emotion profile was as likely as remaining in the rather positive emotion profile (*OR* = 0.77, 95% CI [0.47, 1.26]). However, in Grade 8, the opposite trend was found: We found a higher probability of transitioning from the predominantly positive to the rather positive emotion profile (44.6%), marking an increase in negative mathematics emotions. This transition was as likely as remaining in the predominantly positive emotion profile (*OR* = 0.93, 95% CI [0.57, 1.51]).

### Intervention effects on transition

To test the impact of the intervention on students’ transitions between emotion profiles, we used the control group as a reference. Our results showed that membership in an intervention group (student–teacher or student-only intervention group) had no significant impact on the transition probability compared to the control group. Thus, the likelihood of changing profiles was equally high in the intervention groups and the control group.

**Fig. 3:**
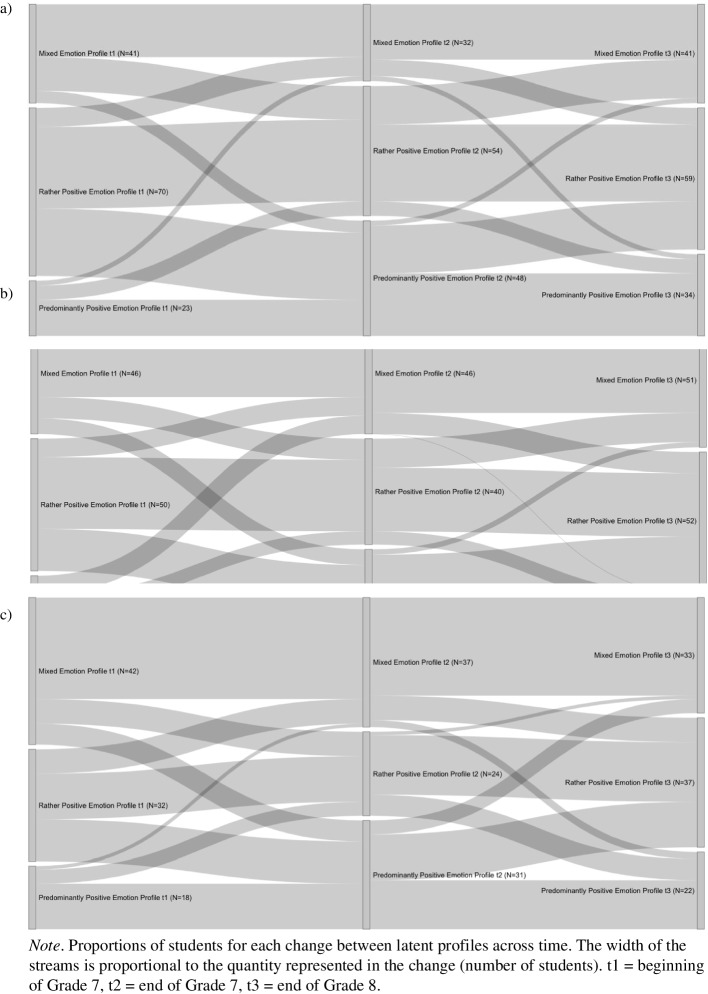
Transitions for **a** student–teacher intervention group, **b** student intervention group, and **c** control group. Note. Proportions of students for each change between latent profiles across time. The width of the streams is proportional to the quantity represented in the change (number of students). t1 = beginning of Grade 7, t2 = end of Grade 7, t3 = end of Grade 8

To illustrate the transitions for each of the three groups separately, intervention group status was included as a grouping variable. The final class counts based on the most likely latent class pattern for each group are displayed in Fig. 3. These Sankey diagrams show that—similar to the overall transition probabilities—there was a positive trend in Grade 7 among all groups. Students tended to transition from the rather positive to the predominantly positive emotion profile (indicating a decrease in negative and an increase in positive emotions). Also, the opposite trend in Grade 8 (transitioning from the predominantly positive to the rather positive emotion profile) is depicted for all groups.

In addition, the odds of transitioning to another profile compared to having the same profile among each group show additional interesting results: In the student–teacher intervention group, transitioning from the mixed to the rather positive emotion profile between the first and second measurement points (*OR* = 0.55, 95% CI [0.24, 1.26]) or the second and third measurement points (*OR* = 0.34, 95% CI [0.10, 1.19]) was not significantly less likely than retaining the mixed emotion profile. In contrast, students in the student intervention group (*OR*_t1-t2_ = 0.286, 95% CI_t1-t2_ [0.11, 0.72]; *OR*_t2-t3_ = 0.24, 95% CI_t2-t3_ [0.08, 0.70]) and the control group (*OR*_t1-t2_ = 0.25, 95% CI_t1-t2_ [0.10, 0.63]; *OR*_t2-t3_ = 0.23, 95% CI_t2-t3_ [0.06, 0.96]) were significantly less likely to transition from the mixed to the rather positive emotion profile than to retain the mixed emotion profile.

Moreover, during Grade 7, students in the student–teacher intervention group were significantly less likely to transition from the predominantly positive to the mixed emotion profile (*OR* = 0.13, 95% CI [0.02, 0.96]). In contrast, students in the student intervention group (*OR* = 0.48, 95% CI [0.17, 1.40]) and students in the control group (*OR* = 0.12, 95% CI [0.01, 1.82]) were equally likely to transition from the predominantly positive to the mixed emotion profile as to maintain the predominantly positive emotion profile.

## Discussion

The major contribution of this study lies in the application of a person-centered approach within a longitudinal intervention study that aims to examine the emotion profiles of students in the lowest-ability tier in lower secondary mathematics education. This design has enabled us to identify emotion profiles, investigate changes in these profiles over two school years, and examine the effectiveness of an intervention in promoting positive mathematics emotions across students with different profiles.

### Mathematics emotion profiles

In line with our expectations, we found three distinct mathematics emotion profiles in the lowest-ability tier in secondary education: a predominantly positive, a rather positive, and a mixed emotion profile. Similar to previous research with other student groups, we identified profiles that were dominated by positive emotions (e.g., Jarrell et al., 2017; Karamarkovich & Rutherford, 2021; Robinson et al., 2020). Also in line with existing results, we found a mixed emotion profile with similar levels of positive and negative emotions (Ganotice et al., 2016). Unexpectedly and in contrast to prior studies (e.g., Jarrell et al., 2017; Karamarkovich & Rutherford, 2021), our sample did not reveal a negative emotion profile. It could have been expected that this profile would be common among our selected sample, which only included students in the lowest-ability tier who have experienced negative selection based on their prior academic achievement. This discrepant finding is encouraging, as existing research has pointed to possible disadvantages for students with a negative emotion profile—for example, regarding motivation or achievement (e.g., Ganotice et al., 2016; Karamarkovich & Rutherford, 2021).

Another encouraging result is the large proportion of students in the rather positive and predominantly positive emotion profiles (62.93–66.95% of all students), as these profiles are positively associated with learning motivation and performance (Ganotice et al., 2016; Karamarkovich & Rutherford, 2021; Robinson et al., 2017). Our findings confirm that the levels of emotion varied between students as well as within students (Martínez-Sierra & García-González, 2017). The mixed emotion profile thereby highlights the possibility that students feel positive and negative mathematics emotions simultaneously.

Within our sample of students in the lowest-ability tier, boys were significantly less likely than girls to have the predominantly positive emotion profile than the other two profiles. This is contrary to existing research that found a larger proportion of boys having a positive profile and more girls having an anxious or resigned profile (Hanin & Van Nieuwenhoven, 2019). However, variable-centered research has also reported that the association between gender and emotions is moderated by mathematics performance. Male secondary school students with low levels of mathematics performance reported more anger, anxiety, and boredom than female students with low levels of performance (Holm et al., 2017). This moderating role of achievement may help to explain our discrepant results, as our study focused on students in the lowest-ability tier. Importantly, we do not claim that gender differences in achievement emotions generalize beyond our specific sample, and further research is needed to investigate whether similar patterns emerge in other educational settings.

Regarding academic achievement, results revealed that students with higher levels in the standardized mathematics test at the beginning of Grade 7 were more likely to show the rather positive or predominantly positive emotion profile. This result is in line with CVT, which suggests that prior mathematics achievement influences emotions (feedback loops; Pekrun, 2006). This highlights that antecedents, emotions, and learning outcomes interact in a dynamic process.

### Stability and change of emotion profiles

One of the main contributions of the present study was to examine the stability of mathematics emotion profiles over two school years. The results of the RI-LTA revealed that student mathematics emotion profiles in the lowest-ability tier in lower secondary education are fairly stable over time, with the majority of students maintaining the same profile. Interestingly, this is in line with previous research with university students that form a rather different population regarding age, academic trajectories, and achievement (Tze et al., 2022). However, there is also a considerable amount of variability in mathematics emotion profiles, indicating that emotion profiles can be changed. Based on existing findings from variable-centered research (e.g., Meyer & Schlesier, 2021; Vierhaus et al., 2016), a decline in positive emotions and an increase in negative emotions in adolescence might be expected over time. The results of our longitudinal study revealed an interesting new developmental pattern: Between the beginning of Grade 7 and the end of Grade 7, we found a positive trend of transitioning from the rather positive to the predominantly positive emotion profile, followed by the opposite trend between the end of Grade 7 and the end of Grade 8. Both forms of transition were equally likely as continuing to have the same profile, whereas all other transitions were significantly less likely.

When testing the effect of the intervention on transitions, no significant difference between the intervention groups and the control group emerged. Thus, we can assume that the transitions were not related to the intervention and might better be explained by structural or social causes. For example, the decrease of negative mathematics emotions during Grade 7 among the students might be explained by the big-fish-little pond effect (Marsh, 1987): Students in Switzerland are assigned to one of three tiers at the end of primary education (Grade 6). In a mixed-ability primary class, students with lower performance in mathematics—like the students in our sample—usually orient themselves upwards. After the transition to a more homogeneous secondary school, however, the frame of reference is likely to change. Students are now at approximately the same academic level, reducing upward comparisons and possibly leading to more positive emotions and less negative emotions (Becker & Neumann, 2018). In Grade 8, however, students in Switzerland face increased pressure to find a position in the labor market or to transition to upper-secondary education, which might lead to an increase in negative emotions (Güntzer, 2017; Nagy et al., 2005) and thus explain the general negative trend. In addition, the curriculum in Grade 8 might play a crucial role: Although the structure of the official teaching material is similar in Grades 7 and 8 (Affolter et al., 2013, 2014), students’ emotions may be influenced by individual content. In addition, as intended by the curriculum, the topics become more demanding over the school years, which in turn can have a negative effect on emotions in mathematics (Hernandez-Martinez & Pampaka, 2017). However, we must also critically note that due to the division into three intervention groups, the number of students per cell and per transition was very low. This may also explain the insignificant effects because smaller sample sizes generate wider confidence intervals (O'Brien & Yi, 2016).

Furthermore, some positive effects in the student–teacher intervention group emerged: Transitioning from the mixed to the rather positive emotion profile was as likely as continuing to have the mixed emotion profile, whereas, in the student intervention and control groups, this transition was significantly less likely. This indicates that the student–teacher intervention had a positive effect on the likelihood of this transition. Another positive effect of the student–teacher intervention can be inferred from the following results: Students in the student–teacher intervention group were significantly less likely to transition from the predominantly positive to the mixed emotion profile during Grade 7, whereas this transition was equally likely as maintaining the predominantly positive emotion profile among those in the student intervention and control groups. These findings highlight the potential added value of involving teachers in the intervention. Teachers in the student–teacher intervention group received dedicated workshops that expanded their knowledge on student emotions and motivation. This increased awareness of emotional and motivational processes may have translated into subtle yet meaningful changes in their teaching practices and classroom interactions. Research suggests that teachers’ emotional support and instructional practices play a critical role in shaping students’ emotions (Frenzel et al., 2009; Lazarides & Buchholz, 2019). Thus, even if the teacher-focused component of the intervention did not explicitly aim to alter instructional methods, possible indirect effects of enhanced teacher awareness may have contributed to the observed differences in emotion profile transitions.

In addition, these findings underscore the importance of tailored interventions (e.g., Cohen et al., 2017), as the intervention was found to have positive effects on some students, especially those within the mixed emotion profile. Interventions that target all students equally might have a limited impact. Rather, tailored interventions might be more effective, and intervention studies that consider different emotion profiles might better contribute to our understanding of student emotions. Overall, more research on individual or group-specific emotional trajectories is needed to understand the differential effects of interventions and their interactions with individual traits (Binning & Browman, 2020; Lapka et al., 2011; Zhao, 2017). Furthermore, contextual effects such as teaching behavior, instructional quality, and classroom climate should be further investigated, as these factors may influence students’ emotional transitions and the effectiveness of interventions. Future research should explore how specific teaching practices, classroom norms, and student–teacher interactions contribute to shaping students’ achievement emotions over time, as well as how teacher-focused interventions can be optimized to maximize their impact on students’ emotional development in mathematics learning.

### Limitations and future directions

Although our longitudinal study extended the existing research on emotion profiles, several limitations need to be addressed. First, we only examined emotion profiles and transitions in the domain of mathematics among lower secondary school students in the lowest-ability tier. Emotion profiles might vary across school subjects, school levels, and ability tiers. A study with a larger and more diverse sample would be needed to validate the profiles identified in this study in terms of other subjects, students, and ability levels.

Second, our profiles are based on five mathematics emotions. The results are, therefore, only partially comparable with other studies. Although these emotions have been identified as basic emotions (Ekman & Cordaro, 2011; Izard, 1992), adding further emotions might lead to more or qualitatively different profiles. In addition, mathematics achievement was measured only at the beginning of Grade 7 using a standardized mathematics test. It remains, therefore, unclear whether the association between mathematics achievement and profile membership would have been maintained at later measurement points. In addition, our study is solely based on quantitative data. Qualitative data, which could have provided deeper insights into students’ experiences and the contextual factors influencing their emotions in mathematics, would have enriched the analysis. Future research should consider combining quantitative and qualitative approaches to explore students’ emotion profiles in more depth.

Third, due to the small sample size, the investigation of the impact of the intervention on transitions was not robust (O'Brien & Yi, 2016). Future studies with larger sample sizes are needed to investigate the possible effects of interventions on transitions. In addition, it cannot be completely excluded that other factors (e.g., personal or environmental factors) may have contributed to the changes in mathematics emotion profiles.

Fourth, the lack of randomization of the three groups must be considered. Although the teachers of the three groups did not differ in terms of their self-reported enjoyment of teaching mathematics (see Brandenberger et al., 2018), it cannot be excluded that the self-selection of teachers might have influenced the results. Thus, future research with randomized assignment is needed to validate the results.

Finally, we did not systematically measure the outcomes of the intervention on teachers (e.g., changes in instructional strategies or attitudes) and the effect of teacher participation in workshops on students. This decision was made for two key reasons. First, the small number of participating teachers made statistical comparisons between groups impractical. Second, we aimed to minimize additional pressure on teachers by avoiding extra assessments that could have influenced their engagement with the intervention. While teachers in one of the intervention groups participated in workshops designed to expand their knowledge of student emotions and motivation, our primary focus remained on the students’ emotional development and the effects of the intervention on their achievement emotions. Future research should further investigate how instructional design or teacher support influences students’ emotional experiences in mathematics education.

### Implications

The present research has several theoretical and practical implications: The study suggests that different emotions co-exist, and students in the lowest-ability tier in lower secondary education in mathematics reveal different emotion patterns. This is an important group for educational research because students allocated in the lowest-ability tier are a special at-risk group for negative emotions (e.g., Pekrun et al., 2017). Unexpectedly, no negative emotion profile was found. Instead, our study revealed that students in the lowest-ability tier can show similar mathematics emotion profiles to students in other ability tiers and other domains (e.g., Ganotice et al., 2016). Further research is necessary to investigate how students cope with negative achievement experiences and how teachers succeed in maintaining students’ positive emotions despite negative achievement experiences. Our results also revealed a mixed emotion profile that shows similar degrees of positive and negative emotions within a group of 115–129 students. Future research should pay attention to a possible interaction of these concurrent emotions as this interaction may have different outcomes than single emotions. For educational practice, these emotion profiles can be used for targeted support by explicitly addressing students with a mixed mathematics emotion profile. In addition, teachers should pay special attention to their own emotions and to the teaching quality in mathematics classes (e.g., instructional and organizational support; Barnes, 2021). Moreover, identifying mathematics emotion profiles could help teachers adapt their teaching methods to students or support social-emotional learning in their classes.

Moreover, to the best of our knowledge, no longitudinal study has investigated the stability and change of emotion profiles over two school years in lower secondary education. Thus, the results extend and enrich existing research on emotion development in secondary education. The high stability of the mathematics emotion profiles also points to the relevance of preventing negative emotion profiles. Both research and educational practice should aim to avoid negative emotional development, as a negative profile remains relatively stable. However, the potential positive intervention effects as well as the high stability of positive mathematics emotion profiles also underscore the relevance of emotion interventions. A targeted intervention may show long-lasting effects on the emotion profile and may accordingly have a large effect on the academic learning of individual students.

## Supplementary Information

Below is the link to the electronic supplementary material.

Supplementary file1 (DOCX 21 KB)

Supplementary file2 (DOCX 19 KB)

Supplementary file3 (DOCX 20 KB)

Supplementary file4 (DOCX 20 KB)

Supplementary file5 (DOCX 17 KB)

## Data Availability

Data and material will be made available on request.

## Footnotes

1 In the majority of the Swiss cantons, students are allocated into three different school tiers after primary school (Grade 6). The main tier allocation criterion is the assessment of academic achievement in societal language, first foreign language, and mathematics. In addition, teacher assessment of non-cognitive competencies (e.g., social competencies) and parental preferences may play a role (Held & Hascher, 2023a).
