# Supplementary material for: Mathematics emotion profiles: stability and change during Grades 7 and 8
Source: Eur J Psychol Educ. 2025 Jun 11;40(2):68. doi: 10.1007/s10212-025-00972-4 (PMC12158859; doi:10.1007/s10212-025-00972-4)
Supplement: Supplementary file 1 — Supplementary file1 (DOCX 21 KB) [file 10212_2025_972_MOESM1_ESM.docx]

**Supplementary Information A**

*Descriptive Statistics and Correlations for Study Variables*

|  |  | *M* | *SD* | 1 | 2 | 3 | 4 | 5 | 6 | 7 | 8 | 9 | 10 | 11 | 12 | 13 | 14 | 15 |
| --- | --- | --- | --- | --- | --- | --- | --- | --- | --- | --- | --- | --- | --- | --- | --- | --- | --- | --- |
| 1 | enjoyment t1 | 3.04 | .98 | 1 | .625*** | .441*** | .727*** | .409*** | .318*** | -.480*** | -.342*** | -.195*** | -.326*** | -.197*** | -.174*** | -.663*** | -.377*** | -.316*** |
| 2 | enjoyment t2 | 3.06 | .91 |  | 1 | .623*** | .478*** | .765*** | .440*** | -.412*** | -.676*** | -.389*** | -.280*** | -.420*** | -.293*** | -.486*** | -.680*** | -.520*** |
| 3 | enjoyment t3 | 3.05 | .75 |  |  | 1 | .413*** | .446*** | .710*** | -.330*** | -.527*** | -.609*** | -.257*** | -.328*** | -.305*** | -.356*** | -.507*** | -.760*** |
| 4 | pride t1 | 3.37 | .96 |  |  |  | 1 | .524*** | .477*** | -.387*** | -.251*** | -.263*** | -.336*** | -.174*** | -.185*** | -.521*** | -.295*** | -.282*** |
| 5 | pride t2 | 3.27 | .90 |  |  |  |  | 1 | .626*** | -.347*** | -.551*** | -.450*** | -.270*** | -.399*** | -.298*** | -.352*** | -.479*** | -.381*** |
| 6 | pride t3 | 3.36 | .76 |  |  |  |  |  | 1 | -.151*** | -.303*** | -.527*** | -.159** | -.251*** | -.312*** | -.324*** | -.401*** | -.580*** |
| 7 | anger t1 | 1.94 | .77 |  |  |  |  |  |  | 1 | .490*** | .383*** | .885*** | .471*** | .340*** | .880*** | .498*** | .323*** |
| 8 | anger t2 | 1.79 | .76 |  |  |  |  |  |  |  | 1 | .611*** | .392*** | .719*** | .436*** | .446*** | .959*** | .638*** |
| 9 | anger t3 | 1.88 | .69 |  |  |  |  |  |  |  |  | 1 | .302*** | .436*** | .727*** | .334*** | .543*** | .905*** |
| 10 | anxiety t1 | 1.98 | .81 |  |  |  |  |  |  |  |  |  | 1 | .498*** | .405*** | .689*** | .373*** | .230*** |
| 11 | anxiety t2 | 1.68 | .62 |  |  |  |  |  |  |  |  |  |  | 1 | .530*** | .394*** | .599*** | .333*** |
| 12 | anxiety t3 | 1.67 | .56 |  |  |  |  |  |  |  |  |  |  |  | 1 | .360*** | .345*** | .546*** |
| 13 | boredom t1 | 2.10 | .76 |  |  |  |  |  |  |  |  |  |  |  |  | 1 | .526*** | .378*** |
| 14 | boredom t2 | 2.06 | .87 |  |  |  |  |  |  |  |  |  |  |  |  |  | 1 | .653*** |
| 15 | boredom t3 | 2.10 | .79 |  |  |  |  |  |  |  |  |  |  |  |  |  |  | 1 |

*Notes.* Range 1 to 5. *p <0.05, **p <0.01, ***p ≤0.01. t1 = beginning of Grade 7, t2 = end of Grade 7, t3 = end of Grade 8.
