# Supplementary material for: Mathematics emotion profiles: stability and change during Grades 7 and 8
Source: Eur J Psychol Educ. 2025 Jun 11;40(2):68. doi: 10.1007/s10212-025-00972-4 (PMC12158859; doi:10.1007/s10212-025-00972-4)
Supplement: Supplementary file 2 — Supplementary file2 (DOCX 19 KB) [file 10212_2025_972_MOESM2_ESM.docx]

**Supplementary Information B**

*Longitudinal Measurement Invariance*

| Model | χ² | df | χ²/df | RMSEA | CFI | ΔRMSEA | ΔCFI |
| --- | --- | --- | --- | --- | --- | --- | --- |
| *Enjoyment* | |  |  |  |  |  |  |
| 1 Configural | 69.75 | 39 | 1.79 | .048 | .984 |  |  |
| 2 Metric | 74.30 | 45 | 1.65 | .043 | .985 | .005 | -.001 |
| 3 Scalar | 80.96 | 51 | 1.59 | .041 | .985 | .002 | .000 |
| 4 Residual | 78.57 | 59 | 1.33 | .031 | .99 | .01 | -.005 |
| *Pride* |  |  |  |  |  |  |  |
| 1 Configural | 8.26 | 6 | 1.38 | .033 | .996 |  |  |
| 2 Metric | 10.48 | 8 | 1.31 | .030 | .996 | .003 | .000 |
| 3 Scalar | 14.22 | 10 | 1.42 | .035 | .993 | -.005 | .003 |
| 4 Residual | 17.03 | 14 | 1.22 | .025 | .995 | .01 | -.002 |
| *Anger* | |  |  |  |  |  |  |
| 1 Configural | 56.16 | 39 | 1.44 | .036 | .984 |  |  |
| 2 Metric | 67.41 | 45 | 1.50 | .038 | .979 | -.002 | .005 |
| 3 Scalar | 83.79 | 51 | 1.64 | .043 | .970 | -.005 | .009 |
| 4 Residual | 90.12 | 59 | 1.53 | .039 | .971 | .004 | -.001 |
| *Anxiety* |  |  |  |  |  |  |  |
| 1 Configural | 88.52 | 72 | 1.23 | .026 | .989 |  |  |
| 2 Metric | 103.83 | 80 | 1.30 | .029 | .985 | -.003 | .004 |
| 3 Scalar | 116.70 | 87 | 1.34 | .031 | .981 | -.002 | .004 |
| 4 Residual | 202.92 | 97 | 2.09 | .056 | .932 | -.025 | .049 |
| *Boredom* |  |  |  |  |  |  |  |
| 1 Configural | 24.04 | 15 | 1.60 | .042 | .988 |  |  |
| 2 Metric | 24.63 | 19 | 1.30 | .029 | .993 | .013 | .003 |
| 3 Scalar | 30.32 | 23 | 1.32 | .030 | .990 | -.001 | .003 |
| 4 Residual | 38.21 | 29 | 1.32 | .030 | .988 | .000 | .002 |

*Notes:* df: Degrees of freedom; CFI = comparative fit index; RMSEA = root-mean-square error of approximation.
