# Supplementary material for: Mathematics emotion profiles: stability and change during Grades 7 and 8
Source: Eur J Psychol Educ. 2025 Jun 11;40(2):68. doi: 10.1007/s10212-025-00972-4 (PMC12158859; doi:10.1007/s10212-025-00972-4)
Supplement: Supplementary file 3 — Supplementary file3 (DOCX 20 KB) [file 10212_2025_972_MOESM3_ESM.docx]

# Supplementary Information C

*Model Fit Criteria of the Two to Six Class Solutions in Latent Profile Analysis Over Three Measurement Points*

| Measurement Point | Number of Profiles | LL | | df | BIC | aBIC | CAIC | Entropy |
| --- | --- | --- | --- | --- | --- | --- | --- | --- |
| t1 | 2 | -1846.55 | | 16 | 3786.74 | 3735.98 | 3802.74 | 0.89 |
|  | 3 | -1662.59 | | 22 | 3453.93 | 3384.14 | 3475.93 | 0.89 |
|  | 4 | -1570.61 | | 28 | 3305.08 | 3216.26 | 3333.08 | 0.93 |
|  | 5 | -1518.08 | | 34 | 3235.13 | 3127.27 | 3269.13 | 0.93 |
|  | 6 | -1479.96 | | 40 | 3194.02 | 3067.12 | 3234.02 | 0.93 |
| t2 | 2 | -1689.69 | | 16 | 3473.02 | 3422.26 | 3489.02 | 0.92 |
|  | 3 | -1514.65 | | 22 | 3158.05 | 3088.26 | 3180.05 | 0.92 |
|  | 4 | -1415.16 | | 28 | 2994.19 | 2905.36 | 3022.19 | 0.94 |
|  | 5 | -1356.26 | | 34 | 2911.50 | 2803.64 | 2945.50 | 0.89 |
|  | 6 | -1178.07 | | 40 | 2590.22 | 2708.13 | 2875.02 | 0.90 |
| t3 | 2 | -1486.65 | | 16 | 3066.93 | 3016.17 | 3082.93 | 0.89 |
|  | 3 | -1352.66 | | 22 | 2834.07 | 2764.28 | 2856.07 | 0.90 |
|  | 4 | -1267.57 | | 28 | 2699.01 | 2610.18 | 2727.01 | 0.91 |
|  | 5 | -1207.89 | | 34 | 2614.75 | 2506.89 | 2648.75 | 0.88 |
|  | 6 | -1300.47 | | 40 | 2835.02 | 2463.33 | 2630.22 | 0.84 |
| Model | | |  | |  |  |  |  |
| Configural Similarity | | | 96 | | 8673.70 | 8269.16 | 8769.70 | 0.90 |
| Structural Similarity | | | 66 | | 8609.34 | 8399.97 | 8675.34 | 0.89 |
| Dispersion Similarity | | | 36 | | 8451.59 | 8476.07 | 8487.59 | 0.88 |
| Distributional Similarity | | | 32 | | 8581.12 | 8479.60 | 8613.12 | 0.88 |

*Note:* LL: Loglikelihood; df: Degrees of freedom; BIC: Bayesian information criterion; aBIC: Sample-size-adjusted Bayesian information criterion; CAIC: Consistent Akaike information criterion (CAIC); t1 = beginning of Grade 7, t2 = end of Grade 7, t3 = end of Grade 8.
