# Supplementary material for: Mathematics emotion profiles: stability and change during Grades 7 and 8
Source: Eur J Psychol Educ. 2025 Jun 11;40(2):68. doi: 10.1007/s10212-025-00972-4 (PMC12158859; doi:10.1007/s10212-025-00972-4)
Supplement: Supplementary file 4 — Supplementary file4 (DOCX 20 KB) [file 10212_2025_972_MOESM4_ESM.docx]

# Supplementary Information D

*Elbow Plots with BIC and CAIC Values*

*Notes:* BIC: Bayesian information criterion; CAIC: Consistent Akaike information criterion (CAIC); t1 = beginning of Grade 7, t2 = end of Grade 7, t3 = end of Grade 8.
