# Supplementary material for: Mathematics emotion profiles: stability and change during Grades 7 and 8
Source: Eur J Psychol Educ. 2025 Jun 11;40(2):68. doi: 10.1007/s10212-025-00972-4 (PMC12158859; doi:10.1007/s10212-025-00972-4)
Supplement: Supplementary file 5 — Supplementary file5 (DOCX 17 KB) [file 10212_2025_972_MOESM5_ESM.docx]

# Supplementary Information E

*Means and Standard Deviations of Variables per Latent Profile*

|  | Mixed emotion profile | Rather positive emotion profile | Predominantly positive emotion profile |
| --- | --- | --- | --- |
|  | *M* (*SD*) | *M* (*SD*) | *M* (*SD*) |
| Enjoyment | 2.53 (0.56) | 3.16 (0.48) | 3.62 (0.57) |
| Pride | 2.92 (0.65) | 3.44 (0.34) | 3.74 (0.64) |
| Anger | 2.65 (0.55) | 1.62 (0.27) | 1.14 (0.11) |
| Anxiety | 2.40 (0.74) | 1.57 (0.30) | 1.22 (0.07) |
| Boredom | 2.88 (0.57) | 1.86 (0.34) | 1.30 (0.15) |
| *n*_t1_ | 129 | 152 | 67 |
| *n*_t2_ | 115 | 118 | 115 |
| *n*_t3_ | 125 | 148 | 75 |

*Notes.* Range 1 to 5. t1 = beginning of Grade 7, t2 = end of Grade 7, t3 = end of Grade 8.
